# Polymer-Drug Anti-Thrombogenic and Hemocompatible Coatings as Surface Modifications

**DOI:** 10.3390/pharmaceutics16030432

**Published:** 2024-03-21

**Authors:** Barbara Zawidlak-Węgrzyńska, Joanna Rydz, Marta Musioł, Aneta Radziwon-Balicka

**Affiliations:** 1 Department of Chemistry, Faculty of Medicine in Zabrze, Academy of Silesia in Katowice, 40-555 Katowice, Poland; 2 Centre of Polymer and Carbon Materials, Polish Academy of Sciences, 41-819 Zabrze, Poland; jrydz@cmpw-pan.pl (J.R.); mmusiol@cmpw-pan.pl (M.M.); 3 Department of Respiratory and Infectious Diseases, Center for Translational Research, Bispebjerg Hospital, University of Copenhagen, 1172 København, Denmark; aneta.radziwon@gmail.com

**Keywords:** surface modification, drug, anti-thrombogenic surface

## Abstract

Since the 1960s, efforts have been made to develop new technologies to eliminate the risk of thrombosis in medical devices that come into contact with blood. Preventing thrombosis resulting from the contact of a medical device, such as an implant, with blood is a challenge due to the high mortality rate of patients and the high cost of medical care. To this end, various types of biomaterials coated with polymer-drug layers are being designed to reduce their thrombogenicity and improve their hemocompatibility. This review presents the latest developments in the use of polymer-drug systems to produce anti-thrombogenic surfaces in medical devices in contact with blood, such as stents, catheters, blood pumps, heart valves, artificial lungs, blood vessels, blood oxygenators, and various types of tubing (such as for hemodialysis) as well as microfluidic devices. This paper presents research directions and potential clinical applications, emphasizing the importance of continued progress and innovation in the field.

## 1. Introduction

Medical devices that come into contact with blood are an important part of modern medicine. In clinical practice, such devices can serve a patient for hours, days, years, or even support the patient’s body for a lifetime. For example, coronary stents and heart valves remain in a patient’s body for many years [1]. Ventricular assist devices are typically implanted either for a short period of time, such as a few weeks or months, as a bridge to a heart transplant, or for a longer period of time, for years, as a target therapy to support or replace the natural function of the heart throughout life. On the other hand, dialysis and extracorporeal circulation circuits have the shortest contact time with blood, usually only during therapeutic procedures. Regardless of the purpose of the medical device or its duration of use, they cause undesirable phenomena when they are in contact with blood. During contact between blood and the endothelium-deprived surface of a biomaterial, adverse processes of activation, adhesion, release, and biosynthesis of vascular active platelet mediators occur. Activation of the blood defense mechanisms is one of the key mechanisms that can adversely affect the performance of medical devices that come into contact with a patient’s blood [2]. To prevent such reactions, drug therapy with antiplatelet drugs or anticoagulants is used, but these can cause postoperative bleeding. Therefore, various methods are being sought to improve the compatibility of biomaterial surfaces with blood. One method of preventing undesirable phenomena when blood interacts with medical devices or biomaterials is surface modification by coating the surface of biomaterials with polymer-drug layers. From simple coatings to advanced functionalized composite coatings, they provide high functionality and can be used on various materials, including metals, ceramics, or polymers. They can play a key role in developing next-generation biomaterials and instruments in medical devices, providing corrosion protection, increasing wear resistance, improving biocompatibility, and can also be used as smart materials. Polymeric coatings, due to their flexibility and ability to be functionalized, are used in many practical applications, including as biomimetic surfaces and for drug release control. The development of advanced coatings requires precise selection of materials, production methods, and parameters to ensure a high performance and appropriate properties [3]. Surface modification with a polymer-drug layer is used to help prevent issues such as thrombosis or biofouling, providing a protective layer that inhibits blood clotting or bacterial adhesion by releasing drugs that have anticoagulant or antimicrobial properties by the polymer-drug layer [4]. Both natural and synthetic polymers due to favorable surface and bulk properties are highly versatile materials that have played an integral role in the development of drug delivery technologies. They have made it possible to achieve controlled release of drugs, ensuring that therapeutic agents are released in consistent doses over extended periods and enabling cyclic dosages. Moreover, their unique properties enable tunable release of both hydrophilic and hydrophobic drugs, further enhancing the effectiveness of drug delivery systems [5,6]. It is therefore worth pursuing improvements in polymer coating manufacturing techniques, which can significantly impact the development of next-generation biomaterials and medical devices.

This review provides a thorough and current overview of surface modifications in medical devices, encompassing existing polymer-drug coatings and those still in the developmental stages.

## 2. Blood Interactions with Biomaterials

The use of blood-contacting biomedical devices such as stents, grafts, heart valves, drug delivery systems, and extracorporeal devices has increased during the last decade. These devices usually cause hemostasis disorders in the vasculature, often depending on the composition of the device. Moreover, the surface of such devices does not have anti-inflammatory or anticoagulant (antithrombotic) properties like the endothelium, but it may have procoagulant properties. In clinical practice, this is solved by treating patients with anticoagulant drugs, which are linked to increased bleeding risks. Therefore, covering the surface of biomedical devices with different materials to reduce blood activation is crucial to decrease the usage of systematic treatment.

Plasma proteins are adsorbed onto the surface of biomaterials from the moment they are exposed to plasma. The layer of proteins is called a protein corona, and their composition depends on the type of biomaterial [7]. Moreover, Jaffer et al. demonstrated that the concentration of the protein corona may be more than 1000-fold higher compared with the plasma concentration [8]. There are several different proteins which play a role in building the corona, such as complement proteins, albumin, vitronectin, fibronectin, fibrinogen, and coagulation factors XI and XIIa [9,10]. This innate event creates the appropriate microenvironment for further activation of coagulation and recruitment of platelets and leukocytes.

The complement system (also complement cascade) is a plasma protein system that plays a crucial role in the immune system by recognizing and effectively defending against pathogens. It can be activated directly by pathogens themselves or indirectly by antibodies bound to pathogens, resulting in a cascade of reactions occurring on the surface of pathogens and generating active components with different effector functions (induction of phagocytosis by phagocytic leukocytes and antibody-dependent cellular cytotoxicity) [11,12]. The complement system is activated via the classic, mannan-binding lectin or alternative pathways [13]. The activation links disposing of foreign materials and damaged cells with the recruitment of inflammatory cells and the initiation healing, which initiates thromboinflammation [14]. The formation of a protease called a C3 convertase and the cleavage of C3 into complement fragments C3b and C3a is a key step in the activation of the complement system. C3b acts as an opsonin to promote phagocytosis, while C3a acts as a potent mediator of inflammation. C3b also binds to C3 convertase to form C5 convertase. This C5 convertase produces C5a, a significant peptide mediator of inflammation, as well as C5b, which plays an important role in initiating the later steps of complement activation [12]. C3a and C5a are important for recruitment and activation of platelets, neutrophiles, and monocytes. In addition, they provoke secretion of von Willebrand factor (vWF), glycoprotein in the blood that promotes hemostasis (particularly platelet adhesion), and P-selectin (transmembrane adhesion receptor) from platelets [15,16].

Plasma contact systems such as contact activation and the kallikrein-kinin system are responsible for triggering coagulation, inflammation, and innate immunity at the blood-biomaterial interface. Within seconds of surface exposure, factor XII (80 kDa glycoprotein synthesized in the liver, which circulates in the blood as a zymogen—an inactive precursor of the enzyme) is activated, which leads to thrombin generation via an intrinsic pathway. The generation of thrombin results in platelet activation and clot formation [17]. In addition, thrombin activates protease-activated receptors on leukocytes [18], which results in the release of proinflammatory cytokines to further recruit platelets and leukocytes. Moreover, Burzynski’s study shows that thrombin can activate complement system to increase inflammation and coagulation through the activation of monocytes [19]. The interaction between platelets and biomaterials is similar to the adhesion of platelets to damaged subendothelium, which is well established.

Platelets are anucleate blood elements derived from megakaryocytes. Their main function is to maintain vascular homeostasis, but they also contribute to thrombosis. During both processes, complex interactions occur between the injured vessel wall and platelets. Platelets adhere to the injured wall, undergo shape changes to form pseudopodia, and release cytokines, chemokines, etc. from their α-granules (typical of platelets and constitute their most numerous granules) and dense δ-granules (lorganelles related to lysosomes that originate from the endosomal compartment), facilitating platelet aggregate/thrombus formation. Platelet adhesion and aggregation reactions are mediated by the principal receptors GPIb/IX/V and GPIIb/IIIa found on the outer surface of the platelet membrane. To prevent an uncontrollable aggregation cascade, an endogenous signaling system exists to limit activation of the receptors. One of the most important platelet inhibitory signaling systems is mediated by nitric oxide (NO). NO is generated by both endothelial cells and platelets [20]. The outer surface of the platelet membrane is very rich in glycoproteins and is known as glycocalyx. The glycoprotein receptors are necessary to facilitate the adhesion, activation, and aggregation of platelets. The glycoprotein GPIb/IX/V and GPIIb/IIIa complexes are the principal mobile receptors on platelets. The outside membrane surface in resting platelets is covered by about 25,000 GPIb/IX/V and 80,000 GPIIb/IIIa receptor copies [21].

Firstly, the GPIb/IX/V complex interacts with vWF. Following damage occurring to the blood vessel, the vWF binds to the subendothelial collagen. vWF plays a major role in blood coagulation, and it is important in platelet adhesion to wounds [22]. The GPIb/IX/V complex includes the GPIbα subunit, which is essential for vWF binding, while GPIbβ and GPIX are responsible for assembling and anchoring the complex to the platelet surface. Furthermore, on the platelet surface, there are two main collagen receptors: GPIa/IIa and GPVI. The main function of GPIa/IIa is to facilitate the bonds between platelets and collagen. GPVI acts as a signaling molecule and fully activates platelets [23].

Secondly, the binding of vWF to the GPIb/IX/X complex leads to the release and activation of GPIIb/IIIa [24,25]. In addition, when the platelets are active, they translocate P-selectin, which is mainly stored in α-granules, to the platelet membrane surface [26,27]. The activation of these receptors leads to a change in the shape of platelets. Also, upon activation, platelets release growth factors and growth regulators such as growth factors, interleukin 6 (IL-6), thrombin, fibrinogen [28], and angiostatin [29,30]. Finally, the circulating soluble fibrinogen and activated platelets form a hemostatic plug that is reinforced by the generation of fibrin by the coagulation cascade, thus forming a thrombus [31].

Systematic therapies are used to prevent thromboinflammation at biomaterial surfaces in clinic practice. Stent thrombosis and recurrent ischemia in patients is managed by antiplatelet drugs such as aspirin and clopidogrel or a P2Y12 inhibitors (clopidogrel, ticlopidine, ticagrelor, prasugrel, and cangrelor) [32]. However, these medications increasing bleeding risks, which indicates the crucial role of platelets.

Therefore, local strategies to cover the biomaterial with anti-thromboinflammatory agents have been investigated. Phosphotidycholine polymers, hyaluronic acid, hydrophilic polymers, or superhydrophobic modification [33,34] are used to reduce protein adsorption and have indicated promising results in preclinical studies [35]. In vascular grafts, heparin (HEP) reduces thromboinflammation and is used for decades [36,37].

Under basal conditions (e.g., resting, thermoneutral, and fasting states), platelets circulate in the resting state. The endothelium maintains these non-adherent and non-thrombogenic conditions by synthesizing releasable mediators such as prostacyclin and NO, both of which inhibit platelet activation [38]. To prevent an uncontrollable aggregation cascade, endogenous signaling systems exist to limit activation of the receptors. One of the most important platelet inhibitory signaling systems is mediated by NO. NO is generated by both endothelial cells and platelets [39]. NO is generated from *L*-arginine and oxygen (O_2_) by the action of the NO synthase (NOS) enzymes such as endothelial NOS (eNOS), cytokine-inducible NOS (iNOS), and neuronal NOS (nNOS). Previous studies have reported the presence of eNOS and iNOS in human platelets [40]. Radomski et al. demonstrated that NO inhibits platelet adhesion to the endothelium and platelet aggregation [41].

Le et al. demonstrated that vascular stents are covered with statins such as rosuvastatin-load nanofibers [42]. There are different coating agents such as plasminogen, thrombomodulin, and tissue-type plasminogen activator (tPA) [43,44,45]. Biofunctional biomaterial surfaces with the ability to promote endothelization are progressively being expanded [46]. The gene complexes incorporated on the biomaterials are involved in endothelial proliferation and adhesion to stimulate endothelization.

The blood-biomaterial interaction is complex and dynamic. There is a gap between in vitro measurements and in vivo studies, especially on the surface of biomaterials [47]. The cellular and molecular events in vivo (protein adsorption and platelet activation) under bio-rheological conditions are crucial to understanding the process of adsorption or binding of molecules and cells to the biomaterial surface over time. Dondosolla et al. demonstrated the use of intravital microscopy for the design of better biomaterials and possibility of personalized adaptations for different clinical purposes [48]. Advances in biomedical technology such as genomics and proteomics are crucial for a deep understanding of the biochemical and molecular pathway dynamics occurring on the blood-biomaterial interface [49].

## 3. Medical Devices

### 3.1. Stents and Catheters

**Figure 1:**
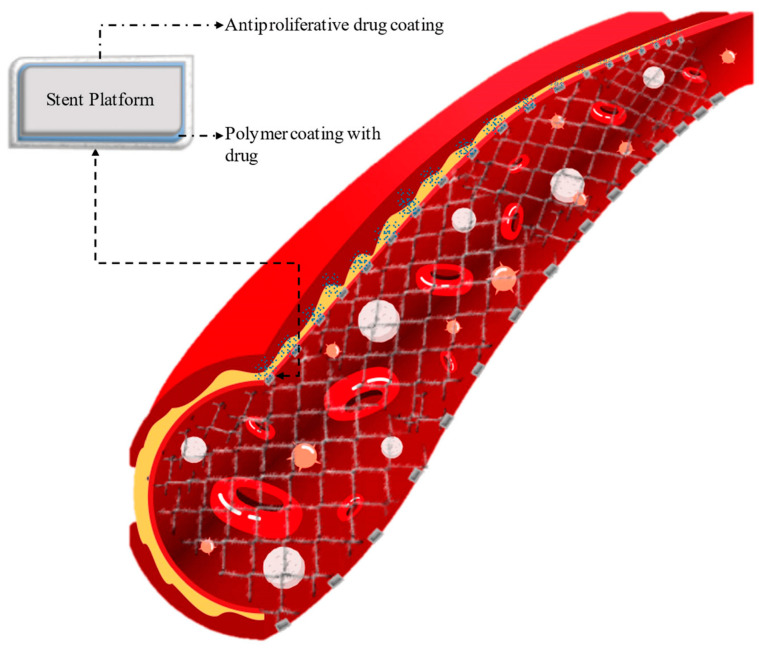
A drug-eluting stent (DES) scheme. Originally published in ref. [52] under CC BY 4.0 license.

Coronary stents are used as a medical device for revascularization of the heart muscle during percutaneous coronary angioplasty. This procedure is important in the case of atherosclerotic cardiovascular disease, which is caused by the deposition of atherosclerotic plaques in the epicardial arteries [50]. Over the years, the design and material from which stents are made have undergone significant modifications. Systematizing the available types of vascular scaffolds, three groups can be distinguished: metal stents—BMSs (bear metal stents), drug-eluting metal stents [51]—DESs (drug-eluting stents) (Figure 1) [52] and a group of bioresorbable stents—BRSs (bioresorbable stents) [53,54].

Metal stents can be made of thin 316L-grade stainless steel, cobalt-chromium, and platinum-chromium. Studies have shown that the use of BMSs, which do not contain antiproliferative drug layers, is associated with high restenosis rates. A breakthrough in coronary artery stent design was the development of DESs [55], which are now widely used due to their superior efficacy compared to BMSs. DESs are the preferred choice for percutaneous revascularization because the release of the drug from the polymer coating helps to reduce the risk of restenosis [56]. These advantages have made DESs the standard treatment for heart disease. The first generation of DESs was constructed of stainless steel and coated with a polymer layer with the drug. Examples of such stents include Cypher (Cordis Corporation, CA, USA), containing a parylene-binding layer and poly(ethylene-*co*-vinyl acetate) (PEVA) and poly(*n*-butyl methacrylate) (PBMA)-releasing sirolimus as well as Taxus (Boston Scientific, Natick, MA, USA) based on poly(styrene-*b*-isobutylene-*b*-styrene) releasing paclitaxel. In the second generation DESs, the stent material was changed to cobalt-chromium or platinum-chromium, and new biocompatible polymers and new antiproliferative drugs (novolimus, zotarolimus, everolimus) were used [57].

Initially, coatings on drug-eluting stents were made from non-degradable polymers such as poly(ethylene-*co*-vinyl acetate), poly(*n*-butyl methacrylate), and poly(styrene-*b*-isobutylene-*b*-styrene). However, these polymers caused inflammation around the implant. Drug release from such coatings was also a problem; for example, a stainless steel implant coated with a paclitaxel-releasing coating released only 10% of the drug contained in the coating. To overcome these problems, non-degradable polymers were replaced by biodegradable polymers. Complete drug elution from the stent coating and a reduced inflammatory response were observed with the use of these polymers [58].

The purpose of using biodegradable polymer coatings is to release the drug once the stent is in place in the human body. Understanding the factors affecting the timing of drug release and the rate of polymer degradation in stent coatings is an important aspect to optimize treatment and achieve better healing outcomes after implantation. Faster elution of the antiproliferative agent could potentially facilitate faster and more robust healing in the critical first few months, potentially reducing the risk of later complications [59]. PEGylation of a biodegradable polymer used in a coating that releases the drug into the bloodstream is a promising method for extending the life of such a coating, so the release process can be controlled [60]. Examples of drug elution times in selected stent platforms are shown in Table 1.

The purpose of the BRS is to offer temporary support to the vessel, enabling it to recover and eventually return to its original and healthy condition. Once its job is done, the BRS will gradually fade away, allowing the vessel to regain its natural strength without any further assistance. This transition is crucial to ensure the vessel’s long-term well-being and sustained functionality. BRS is typically made of a biocompatible polymer material that is inserted into the blood vessel to help keep it open and improve blood flow. They provide a scaffold-like support structure and can be tailored to specific patient needs. Polymer stents are often preferred over metal stents due to their flexibility, ease of use, and reduced risk of causing vascular injury. Polyesters are commonly used in BRS technologies because of their tailored biodegradation. These polymers can be designed to degrade at different rates depending on the desired time. Poly(*L*-lactide) (PLLA), poly(*D*,*L*-lactide) (PDLLA), poly(lactide-*co*-glycolide) (PLGA), polycaprolactone (PCL) [66], and polycarbonates have been extensively investigated as possible materials for the development of BRSs [67,68].

BRSs have significant advantages over permanent metal stents. BRSs reduce the risk of thrombosis in later stages, allow for the restoration of vasomotion and the artery’s original shape, and offer the potential for reoperation. PLLA, a biodegradable semicrystalline polymer, is extensively utilized in the medical device industry. This is primarily because of its excellent biocompatibility, processability, controlled degradation, and superior mechanical properties in comparison to other biodegradable polymers, allowing it to perform specific tasks as a stent [69]. However, for PLLA to be suitable for use as a stent, it must fulfill certain criteria regarding its mechanical properties. The processing stents has an influence on their mechanical properties. Biaxial expansion was found to be more suitable for stent application due to the more isotropically distributed crystals in the stretching planes and significantly enhanced mechanical properties. The study showed a positive correlation between the stretch ratio and elastic mechanical properties across the ranges investigated [70].

PDLLA can be used as one element of a stent; for example, it can be applied as a coating with a drug such as sirolimus [71], paclitaxel [72], prasugrel [73], or everolimus [74]. Ultrasonic atomizing spray technology was used for preparation of PDLLA coatings with sirolimus (immunosuppressive drug) on PLLA stents. The spray coating applied to the surface of the stent was found to be uniform and smooth, with a relatively even distribution. The coating appeared to be consistent across the entire stent surface, indicating a high level of accuracy in the application process. Additionally, the coating was discovered to possess exceptional resistance against significant deformations. The release profiles of sirolimus in vitro demonstrated a consistent pattern of biphasic release, characterized by an initial burst period of release followed by sustained release over time. Sirolimus acts as an anti-proliferative drug, preventing the growth of smooth muscle cells that contribute to restenosis. The polymer coating ensures controlled and sustained release of sirolimus, enhancing the stent’s effectiveness in reducing arterial reblockage and improving patient outcomes [75].

PLLA stents were used as a base for coatings applied where a blend of PDLLA and PLGA was adopted. Different compositions of PDLLA/PLGA coatings were used to evaluate the ability of a system or structure to withstand or distribute radial loads or forces applied perpendicular to its axis. In this area, the PDLLA/PLGA (80/20) blend showed the best properties. The study also found that the coatings on the stents exhibited reliable adhesion. These findings offer a more convenient approach to enhancing the mechanical properties of stents [76].

Zhao et al. [77] developed a new method for stent coating. Their study showed that the use of PDLLA nanoparticles loaded with syrolimus could be an effective strategy for the prevention of vascular smooth muscle cell hypertrophy and a reduction in thrombosis risk. The results from cell culture studies suggest that stents coated with these nanoparticles have the potential to reduce restenosis and vascular inflammation, which may be important for improving the efficacy of these medical devices.

Additive manufacturing 3D printing can be used to create BRSs [78]. PCL as a material for stent preparation was examined to define the influence of different processing parameters, such as nozzle temperature, fluid flow rate, etc., on the physical characteristics of the BRSs. The findings of this research provide valuable insights into optimizing the manufacturing process and enhancing the quality of BRS production [79]. Lee et al. used a 3D printer to fabricate a biodegradable stent with PLLA. The surface of the stent was modified with polidopamine (PDA), polyethyleneimine (PEI), and HEP. The results of a biocompatibility study of the resulting stent showed that the PLLA-modified stent demonstrated good compatibility with blood and showed advantages in preventing restenosis and thrombosis with anticoagulation [79,80].

The REVA stent (REVA Medical, San Diego, CA, USA) is made from a polycarbonate derived from tyrosine. This polymer breaks down into amino acids, ethanol, and carbon dioxide (CO_2_). The absorption time of the stent can be adjusted based on the needs of the patient. It is also balloon expandable, which enables secure placement of the stent [81].

However, polycarbonates can also be applied to the zinc (Zn) alloy in the form of coatings that are composite with HEP. The HEP-antithrombin-III (plasma protein) complex inactivates several coagulation enzymes, including thrombin and factors XIa, Xa, and IXa. As a result, the composite coating demonstrated excellent anti-thrombogenic properties, making it a promising material for applications in biomedical devices and implants. Crosslinking using non-toxic Zn-ligand coordination chemistry played a vital role in enhancing the water resistance and mechanical properties of the obtained coatings [82].

In the field of medical devices, polymers are also used for obtaining catheters which possess various functions such as delivering medication, draining fluids, or providing access to perform some medical procedures. They are made from specialized polymer materials that are flexible, durable, and biocompatible, allowing for safe and effective use in the body. These catheters offer advantages like reduced risk of infection, improved patient comfort, and ease of use for healthcare professionals [83,84]. Mulinti et al. developed a new modified spider silk coating containing HEP. Tests conducted on the resulting coating showed that it could be used to coat catheters, providing antimicrobial and anticoagulant properties [85,86]. Liu et al. developed coatings containing sodium heparin with a quaternary organosilicon ammonium surfactant. These coatings were used to coat intravascular catheters. Animal studies have shown that the applied coating can be used to combat catheter-related bloodstream infections and thrombosis [87].

Urinary catheters are the most popular catheters and have been widely used in various medical and surgical settings as an essential medical device. These catheters are crucial for draining urine from the bladder in patients who are unable to do so themselves. They are commonly used in hospitals, nursing homes, and other healthcare facilities to manage urinary retention or to aid in surgical procedures. However, catheter-associated urinary tract infections are frequently acquired by patients in healthcare settings and pose a significant problem. Catheter-associated urinary tract infections are a major source of drug-resistant pathogens, making it challenging to effectively treat such infections. In order to mitigate the risk of catheter-associated urinary tract infections and prevent the spread of antimicrobial resistance, the polymer-based catheter can be coated to facilitate controlled release of antimicrobial peptides. The development of an ethyl cellulose/1-palmitoyl-2-oleoylphosphatidylcholine-based diffusion layer over an antimicrobial peptide-laden PCL base layer allowed for sustained peptide release over a clinically relevant duration. This controlled the diffusion of antimicrobial peptide into the environment, providing effective long-term delivery of the peptide. The study confirms that an ethyl cellulose/1-palmitoyl-2-oleoylphosphatidylcholine/antimicrobial peptide/PCL coating on silicone catheters effectively reduces bacterial infection. This has the potential to enhance clinical outcomes and encourages further research on manufacturing these solutions [88]. Both stents and catheters are important tools in modern healthcare for providing essential medical treatment and interventions.

### 3.2. Blood Pumps and Heart Valves

Blood pumps and heart valves are important medical devices that are used in the field of cardiology. Blood pumps, also known as ventricular assist devices, are used to support or replace the pumping function of a weakened or failing heart. Heart valves, on the other hand, play a crucial role in maintaining the flow of blood through the heart. They ensure that blood flows in the right direction and prevent any backward flow.

The evolution of blood pumps has been a significant development in medical technology. From early design concepts to modern, sophisticated devices, blood pumps have revolutionized the treatment of cardiovascular diseases. The development of pulsatile pumps, first used in the 1950s, paved the way for continuous flow pumps that are now commonly used in heart transplants and as cardiac assist devices. The ongoing advancements in blood pump technology continue to improve patient outcomes and redefine the field of cardiology [89]. Research carried out in this area improves the construction of pumps, which leads to increasingly better results. For example, the STM CP-24 I (Jiangsu STMed Technology Co. Ltd., Suzhou, China) centrifugal pump is highly effective due to its exceptional hydraulic performance, well-designed hemodynamic structure, and superior blood compatibility. It is specifically designed to meet the requirements of medical professionals, ensuring it can fulfill clinical needs effectively [90].

**Figure 2:**
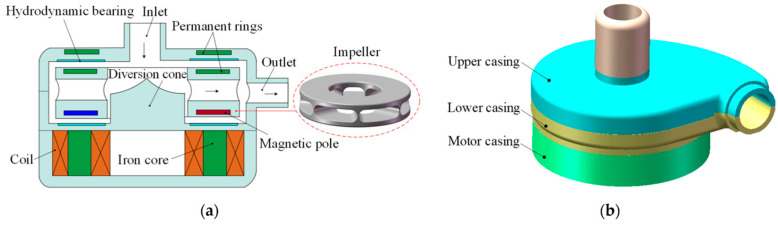
Rotary blood pump: (**a**) schematic of the blood pump design; (**b**) housing model. Originally published in ref. [91] under CC BY 4.0 license.

Biomedical materials used in heart pumps include metals, polymers, ceramics, and composites. These materials need to be biocompatible, and the specific placement of each material within the pump is determined by its intended function. Pulsatile and rotary blood pumps (Figure 2) are the two main directions in this area. Both need to be made of materials that do not elicit negative biological reactions.

Manufacturing artificial organs using nanotechnology materials or polymer composites enhances their adaptability and improves their mechanical properties, biocompatibility, thermal properties, and pump durability [90]. This results in improved efficiency and reduced risk of device failure [92]. Centrifugal blood pumps fabricated using poly(methyl methacrylate) (PMMA) have been created as prototypes. Polydimethylsiloxane, a silicone derivative, has been also used in blood pumps [92].

Another important heart problem is valvular heart disease, which refers to any condition that affects the valves of the heart. The heart has four valves—the aortic valve, mitral valve, tricuspid valve, and pulmonary valve—that open and close to regulate blood flow. When these valves become damaged or diseased, they may not function properly, leading to a disruption in blood flow [93].

The high number of heart valve replacements performed in the United States in 2020 indicates a significant prevalence of heart valve disease and the need for surgical interventions. This highlights the importance of early detection and treatment of heart valve conditions to ensure better cardiovascular health outcomes for patients. Flexible leaflet polymeric heart valves use polymeric materials that are both flexible and durable, allowing them to mimic the function of native heart valves more closely. Polymeric heart valves are a potential solution for heart valve replacement due to their expected durability and lower risk of blood clots compared to bioprosthetic and mechanical valves. Their flexibility allows for minimally invasive implantation procedures, reducing the risks and complications associated with open-heart surgeries. While further research and development are still needed to optimize the performance and long-term durability of these polymeric heart valves, they hold great promise for revolutionizing the field of heart valve replacement therapies [94]. Research on polymeric heart valves frequently uses materials such as polyurethane, polytetrafluoroethylene, poly(styrene-*b*-isobutylene-*b*-styrene), biodegradable elastomers, and hydrogels. These materials offer desirable properties such as biocompatibility, durability, and flexibility that are crucial for the function and longevity of the valve in the dynamic and challenging environment of the human heart [95]. Materials already available on the market are used for research on the development of heart valves, including nanocomposites created by combining functionalized graphene oxide and poly(carbonate-urea)urethane grafts (Hastalex^®^, NanoRegMed Inc., London, UK). The study has confirmed that Hastalex^®^ possesses the necessary attributes for producing artificial heart valves. Its mechanical properties outperform other materials, showing a high compatibility with blood, and it also exhibits resistance against calcification [96].

The innovative manufacturing process of the rotary jet spinning method allows for the production of biomimetic semilunar heart valve scaffolds (JetValve, AIRWOLF Co., Ltd., Xikou, China) with precise control over the fiber diameter and alignment, resulting in a highly functional and durable product. Nanofibers wrap themselves around heart valve-shaped mandrels. The polymeric nanofibers also provide enhanced mechanical properties and biocompatibility, making the JetValve a promising solution for various medical applications. The use of a new polymer material called PLCL has shown promising results. This material, based on connection of PCL and polylactide (PLA), enhances the infiltration of living cells once implanted in the body. Studies on rats have demonstrated that PLCL lasts for around six months, allowing for cellular infiltration and tissue remodeling, then ultimately biodegrading. These findings suggest that heart valves from PLCL obtained via rotary jet spinning could have potential in improving tissue integration and compatibility when used in humans [97].

Three-dimensional printing is also one of the techniques that can be used to obtain artificial heart valves. This technique allows for precise customization and fabrication of valves tailored to individual patient needs, offering the potential for improved outcomes in heart valve replacement surgeries [98].

The development of ink with poly(vinyl alcohol), gelatin, and carrageenan for 3D printing of tissue-engineered heart valves shows promising results. The inks have mechanical properties that are similar to native leaflets, and subcutaneous implantation reveals that they do not cause chronic inflammation and can undergo remodeling. The results of in vitro hemocompatibility assessments indicate that the 3D-printed heart valves exhibit minimal hemolysis and low thrombogenicity. The successful printing of various sizes and types of tissue-engineered heart valves with a high accuracy, along with their ability to withstand aortic conditions as confirmed by hydrodynamic assessments, suggests that these 3D-printed tissue-engineered heart valves hold promise as an alternative for valve replacement, addressing the limitations associated with current options [99].

Hemocompatible polymeric leaflets have been designed and manufactured for polymeric heart valve applications from materials containing interpenetrating networks of hyaluronan and linear low-density polyethylene (LLDPE). This process involved using the solvent infiltration technique and taking advantage of the swelling kinetics of hyaluronan and LLDPE. By introducing hyaluronan into LLDPE, the researchers were able to modify and enhance its properties, potentially leading to improved performance in the proposed applications. The study found that the modified LLDPE materials displayed decreased thrombus formation and platelet activation compared to unmodified LLDPE [100].

The use of poly(vinylidene-*co*-hexafluoropropylene) to create macrovoid-free membranes with optimal pore sizes, combined with a coating of amorphous perfluorinated copolymer of tetrafluoroethylene and 2,2,4-trifluoro-5-trifluorometoxy-1,3-dioxole (Hyflon^®^ AD60X, Solvay Solexis S.p.A., Spinetta Marengo, Italy), resulted in membranes with improved wetting stability. These fluoropolymer-coated membranes exhibited low protein adsorption, high contact angles for water and blood, and performed competitively in blood oxygenation tests using animal sheep blood. Furthermore, there was no detectable hemolysis, making them comparable to commercial polyolefin membranes [101].

Blood pumps and heart valves play a crucial role in supporting and treating patients with impaired cardiac and respiratory functions, improving their overall quality of life and survival chances. Despite numerous improvements and modifications, there is still a need to conduct intensive research to increase patient safety.

### 3.3. Artificial Lungs and Extracorporeal Membrane Oxygenation

**Figure 3:**
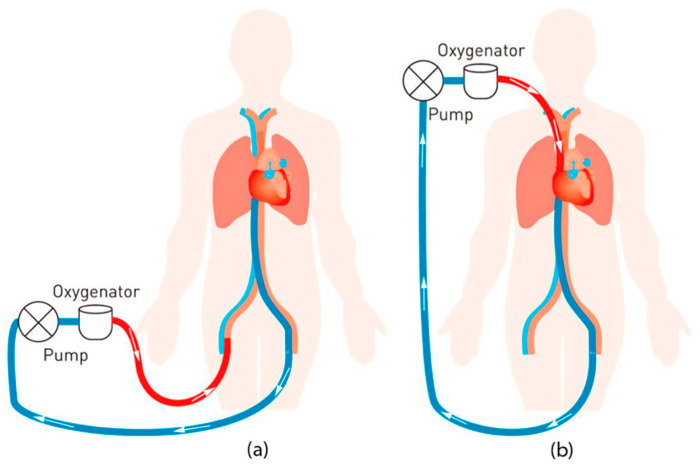
Diagram of the extracorporeal membrane oxygenation model: (**a**) diagram of the veno-arterial system; (**b**) diagram of the veno-venous system. Originally published in ref. [103] under CC BY 4.0 license.

Lung disease continues to be a major health concern in the United States, ranking as the fourth-leading cause of death. It poses a significant threat to public health, contributing to numerous fatalities each year. Respiratory support while waiting for a transplant is still a vast field of research involving the use of mechanical ventilation or extracorporeal membrane oxygenation, but researchers are also striving to develop wearable pumping artificial lungs. Artificial lungs, or extracorporeal membrane oxygenation systems, are used to provide support to patients with severe lung dysfunction, allowing oxygenation of blood outside the body [102]. Extracorporeal membrane oxygenation is a life-saving device that provides temporary support for patients with severe respiratory and circulatory failure. Extracorporeal membrane oxygenation can help improve oxygenation and give the heart and lungs time to recover, but it is a complex and resource-intensive therapy that requires specialized management. Figure 3 shows a schematic model representation of the extracorporeal membrane oxygenation circuit with oxygenated blood in red and deoxygenated blood in blue.

To prevent thrombus formation in patients receiving extracorporeal membrane oxygenation, continuous systemic anticoagulation is necessary due to the high risk of clotting and bleeding. The large surface area and areas of low, turbulent, and stagnant flow in the membrane oxygenator make it particularly susceptible to thrombus formation [104]. Accordingly, several surface coatings have been developed to reduce thrombus formation during extracorporeal membrane oxygenation, with a particular focus on oxygenator coatings. These surface coatings have shown promise in reducing thrombus formation during extracorporeal membrane oxygenation by providing a non-thrombogenic surface that prevents platelet adhesion and activation.

The use of HEP-coated circuits helps to reduce the risk of clot formation and decreases the need for systemic anticoagulation during extracorporeal membrane oxygenation support. This can potentially decrease the risk of bleeding complications and improve patient outcomes. Heparin-coated extracorporeal membrane oxygenation circuits were developed in 1983 as a way to reduce the risk of bleeding and the need for systemic anticoagulation during extracorporeal membrane oxygenation treatment. The HEP coating imitates the anti-thrombogenic effects of heparan sulfate at the endothelium and is stable, releasing only small amounts of HEP into the bloodstream. This coated surface is highly thromboresistant and can prevent clotting even in non-anticoagulated blood. The surface modification of polysulfone (PSF) membranes using plasma treatment and the grafting of poly(ethylene glycol) (PEG) and HEP was investigated to create PSF-PEG-HEP membranes for artificial lung applications [105]. The effects of various plasma treatment parameters were evaluated, and the membranes were characterized using different techniques. The results showed improved hemocompatibility of the modified membranes, as observed through protein adsorption, platelet adhesion, and coagulation tests. Gas exchange tests demonstrated that the PSF-PEG6000-HEP membrane had permeation fluxes of O_2_ and CO_2_ close to the gas exchange capacity of a commercial membrane oxygenator at a blood flow rate of 5.0 L/min. A study by Große-Berkenbusch et al. [106] on the time-dependent adsorption of human plasma proteins on uncoated and HEP-coated oxygenator membranes was designed to determine how protein adsorption differs over time on uncoated and HEP-coated oxygenator membranes. Protein binding was analyzed in loop circuits over 6 h to assess differences in protein adsorption between the two types of membranes. This study provides new insight into the time-dependent adsorption of plasma proteins on uncoated and HEP-coated membranes. It shows that the presence of HEP on the membrane surface modifies protein binding mechanisms and leads to reduced protein binding.

In another study on the modification of oxygenator membranes, a covalent C1-esterase inhibitor (C1-INH) coating was used. The C1-INH protein not only inhibits complement activity but also plays a role in preventing the activation of a zymogen present in the blood that tends to adsorb on the surfaces of medical devices in contact with blood, factor XII, which is an important step in the process of coagulation and inflammation. This dual action of C1-INH makes it a crucial regulator of both the complement system and the coagulation cascade, highlighting its significance in maintaining the balance between these two interconnected pathways. The results of the in vitro study on combined C1-INH/HEP coatings indicate that they are highly effective in preventing platelet adhesion and fibrin networks. This suggests that these coatings have significant potential in preventing clot formation in larger-scale models, such as full-sized oxygenators, where the effect is expected to be even more pronounced [107]. Zhang et al. [108] demonstrated the design of a polydopamine /lysine/HEP composite coating on poly(vinyl chloride) (PVC) tubing to reduce thrombosis by immobilizing anticoagulants and creating bioinert surface strategies. The coating showed significant inhibition of platelet adhesion and activation, prolonged activated partial thromboplastin time, excellent hemocompatibility, and a low hemolysis rate, indicating potential for anticoagulation treatment in medical devices. The composite coating showed promising results in improving the anticoagulant properties of medical materials in an in vitro blood circulation test. The composite coating constructed in this work shows great potential in anticoagulant treatment for new and existing medical devices. Another PVC surface modification involves obtaining a heparinized surface containing sodium alginate and carboxymethyl chitosan. Modified PVC surfaces were obtained using plasma treatment technology and the chemical grafting method. The modification of the PVC surface using this method can be used in the production of pipes in contact with flowing blood in medical devices such as extracorporeal membrane oxygenation [109].

### 3.4. Hemodialysis Membranes

**Figure 4:**
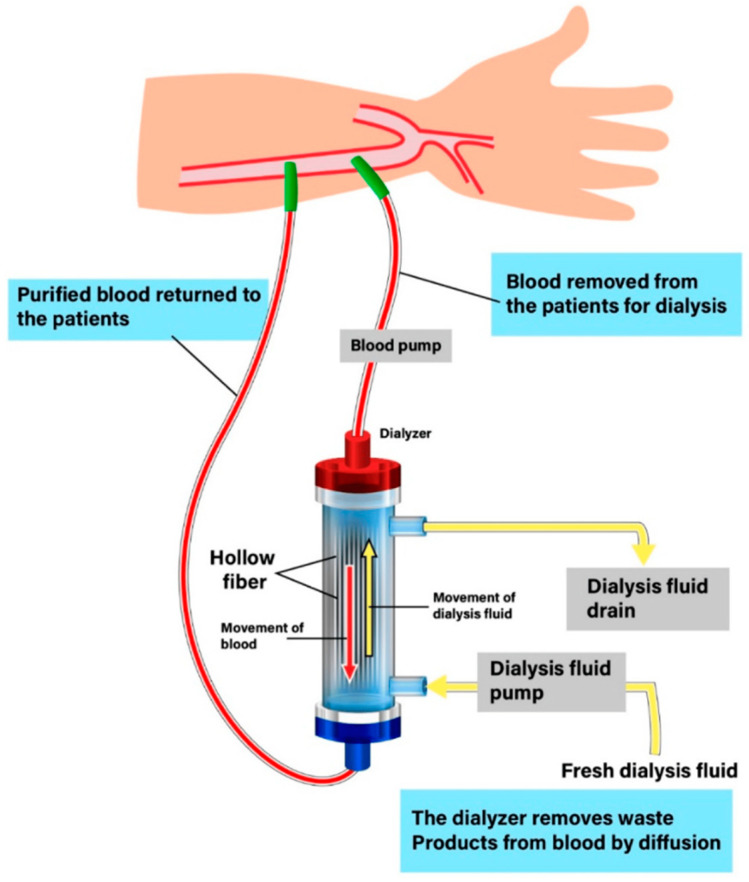
Diagram of the dialysis process. Originally published in ref. [112] under CC BY 4.0 license.

Hemodialysis is a procedure used to treat end-stage renal disease by filtering waste and excess fluid from the blood outside the body [110]. Dialysis membranes are crucial components in artificial kidneys, as they facilitate the removal of various impurities from the blood of patients with kidney disease [111]. These membranes need to possess specific characteristics like good compatibility with the blood, proper permeability, precise selectivity, and consistent performance to effectively perform the dialysis process (Figure 4).

These surface coatings, such as HEP, are effective in improving the blood compatibility of biomaterials by reducing clotting time and minimizing platelet adhesion and protein adsorption [113]. However, despite these improvements, membrane-based therapy still poses significant risks, including acute side effects, chronic conditions, and high morbidity and mortality rates. Therefore, further research and development are needed to find alternative therapies with better safety profiles. A study by Rose et al. [114] highlights the effective use of chitosan to heparinize dialysis membranes, resulting in improved charge and water permeability. The researchers also proposed a simplified, one-step method for manufacturing chitosan membranes, eliminating the need for complex coating procedures. Membranes fabricated using chitosan with a heparinized surface have several important features: significant differences in surface charges, effective resistance to fouling, excellent compatibility with blood, and enhanced permeability to pure water. These findings demonstrate the potential of these membranes for use in hemodialysis. Chitosan was used to immobilize HEP on poly(*N*-isopropylacrylamide) (PNIPAM). PNIPAM is a polymer that is widely used for controlled drug release and for tissue engineering purposes. Ten layers of chitosan and HEP were formed and cross-linked using 1-ethyl-3-(3-dimethylaminopropyl)carbodiimide and *N*-hydroxysuccinimide to increase the stability and reduce the surface charge. The cross-linked complex showed better biocompatibility for C3H10T1/2 cell growth after 24 h in culture [115]. C3H10T1/2 is a cell line exhibiting fibroblast morphology that was isolated from a C3H murine embryonic mesenchymal progenitor cell line, which has been considered as a mesenchymal stem cell model due to its multilineage differentiation potential [116].

## 4. Final Conclusions

Researchers are investigating various methods to improve the compatibility of implant surfaces with blood, as blood’s natural tendency to clot can be a problem when foreign materials are introduced into the body. By increasing the hemocompatibility of implant surfaces, the risk of clots and other complications can be reduced, leading to improved patient outcomes. One avenue of investigation involves developing antifouling and anticoagulant materials using tunable structures with desirable properties, which can potentially outperform HEP in terms of hemocompatibility. Additionally, complex conjugates incorporating HEP may offer a solution, although controlled synthesis of HEP-mimicking polymers appears to be a more promising approach. These advancements in improving hemocompatibility hold the potential to minimize clotting risks and other complications, ultimately leading to improved patient outcomes in biomedical applications. The future prospects for engineering to improve surface hemocompatibility are very promising. Advances in nanotechnology will allow for precise control of coating properties, leading to the development of nanostructured coatings and nanoparticles that enhance biocompatibility and reduce thrombogenicity [117]. Personalized medicine may enable the creation of coatings tailored to individual patients, optimizing treatment outcomes. Biomimetic coatings using bioactive molecules from natural sources and improved drug delivery mechanisms will also be explored. Biodegradable coatings and advanced evaluation techniques will further improve treatment outcomes. Ongoing research in these areas holds great potential for improving the safety and efficacy of implantation and ultimately enhancing the treatment of cardiovascular disease. In summary, the future of medical device coating surface engineering lies in the development of biocompatible and bioactive coatings that mimic the extracellular matrix, contain bioactive molecules, and facilitate cell adhesion and growth. Further research is needed to improve drug delivery efficiency, achieve controlled release profiles, and minimize side effects. Future research should focus on optimizing coating materials, improving drug delivery mechanisms, and conducting thorough biocompatibility and safety assessments to ensure the efficacy and safety of bioactive surface-engineered coatings. In addition to these areas, future research on bioactive surface-modified coatings should also explore their long-term durability and stability, as well as their potential to minimize bacterial adhesion and prevent infection. Moreover, it is important to explore the scalability and cost-effectiveness of manufacturing processes for these coatings to enable their widespread use in medical devices.

## Figures and Tables

**Table 1:** Examples of drug elution rates from stent coatings.

Stent	Drug	Dosage [μg/mm^2^]	Drug Release [%]/Time [week]	Polymer/Coating	References
Alex (Balton, Warsaw, Poland)	Sirolimus	1.0	100/8	poly(*D*,*L*-lactide-*co*-glycolide)	[60,61]
Orsiro (Biotronik, Poznań, Poland)	Sirolimus	1.4	50/4 80/12	PROBIO poly(*L*-lactide)	[61,62,63]
BioMatrix (Biosensors, Irvine, CA, USA)	Biolimus A9™	15.6	45/4	polylacticacid	[63,64]
Ultimaster™ (Terumo Corporation, Tokyo, Japan)	Sirolimus	3.9	100/12–16	poly(*D*,*L*-lactide-*co*-caprolactone)	[64,65]
Synergy™ (Boston Scientific, Marlborough, MA, USA)	Everolimus	1.0	12	poly(*D*,*L*-lactide-*co*-glycolide)	[65]
